# Semi-supervised learning for an improved diagnosis of COVID-19 in CT images

**DOI:** 10.1371/journal.pone.0249450

**Published:** 2021-04-01

**Authors:** Chang Hee Han, Misuk Kim, Jin Tae Kwak

**Affiliations:** 1 Department of Computer Science and Engineering, Sejong University, Seoul, Korea; 2 Department of Data Science, Sejong University, Seoul, Korea; 3 School of Electrical Engineering, Korea University, Seoul, Korea; Liverpool John Moores University, UNITED KINGDOM

## Abstract

Coronavirus disease 2019 (COVID-19) has been spread out all over the world. Although a real-time reverse-transcription polymerase chain reaction (RT-PCR) test has been used as a primary diagnostic tool for COVID-19, the utility of CT based diagnostic tools have been suggested to improve the diagnostic accuracy and reliability. Herein we propose a semi-supervised deep neural network for an improved detection of COVID-19. The proposed method utilizes CT images in a supervised and unsupervised manner to improve the accuracy and robustness of COVID-19 diagnosis. Both labeled and unlabeled CT images are employed. Labeled CT images are used for supervised leaning. Unlabeled CT images are utilized for unsupervised learning in a way that the feature representations are invariant to perturbations in CT images. To systematically evaluate the proposed method, two COVID-19 CT datasets and three public CT datasets with no COVID-19 CT images are employed. In distinguishing COVID-19 from non-COVID-19 CT images, the proposed method achieves an overall accuracy of 99.83%, sensitivity of 0.9286, specificity of 0.9832, and positive predictive value (PPV) of 0.9192. The results are consistent between the COVID-19 challenge dataset and the public CT datasets. For discriminating between COVID-19 and common pneumonia CT images, the proposed method obtains 97.32% accuracy, 0.9971 sensitivity, 0.9598 specificity, and 0.9326 PPV. Moreover, the comparative experiments with respect to supervised learning and training strategies demonstrate that the proposed method is able to improve the diagnostic accuracy and robustness without exhaustive labeling. The proposed semi-supervised method, exploiting both supervised and unsupervised learning, facilitates an accurate and reliable diagnosis for COVID-19, leading to an improved patient care and management.

## Introduction

A global pandemic of coronavirus disease 2019 (COVID-19), declared by the World Health Organization (WHO), has been reported to affect over 19 million patients in >200 countries as of August 9, 2020. COVID-19, caused by the acute respiratory syndrome coronavirus 2 (SARS-CoV-2), is the 7th known coronavirus to infect humans [1], believed to be a zoonotic infection similar to severe acute respiratory syndrome (SARS) and Middle East respiratory syndrome (MERS) [2]. Fever and dry cough are the most common clinical symptoms of the disease [3], similar to many other viral syndromes. Nonspecific symptoms, including dyspnea, headache, muscle soreness, and fatigue, have been also observed. About 20% of the patients experience severe symptoms, and mortality is approximately 3% [4]. Elderly patients, in particular, have a higher chance of experiencing severe symptoms with increased mortality [5]. Therefore, there is a high demand for the immediate diagnosis, treatment, and management of the disease.

A real-time reverse-transcription polymerase chain reaction (RT-PCR) test has been developed and used in clinics for the diagnosis of COVID-19. Even though it serves as the reference standard for the diagnosis, there is a growing number of evidence that CT could replace or complement to RT-PCR test for an improved patient care [6]. Several studies have identified imaging features for COVID-19 on CT [7]. Common CT features include bilateral and peripheral ground-glass and consolidative pulmonary opacities with or without vascular enlargement, interlobular septal thickening, and air bronchogram sign [8, 9]. A longer time of infection has been also associated with more frequent CT findings, including greater total lung involvement, linear opacities, crazy-paving pattern, and the reverse halo sign [9], i.e., CT may be able to monitor and visualize the early, progressive, and severe status of COVID-19. Moreover, CT findings have shown to be effective in identifying COVID-19 for the patients with an initial false-negative result from RT-PCR test [10, 11]. However, the enormous number of cases overwhelms the capacity of hospitals and radiologists, potentially leading to an inaccurate diagnosis. An automated, accurate, objective, and robust computerized system for COVID-19 diagnosis could aid in improving the diagnostic accuracy and yield and patient management and care.

Deep learning has been successfully applied to a wide range of problems in medical image processing and analysis [12, 13]. For example, it can automatically detect abnormalities on head CT scans [14]; diabetic retinopathy and related eye disease can be identified in retinal images with high accuracy [15]; it is also able to detect lymph node metastases [16] as well as segment tissues [17] in pathology images. One of the immediate challenges in deep learning is to obtain a sufficient number of samples with pertinent labels. The capability of deep learning in dealing with a high quantity and quality of samples has been already proved in many applications [18]. Many of previous deep learning methods for medical imaging, in particular, have been mainly developed in a supervised fashion, requiring extensive data annotations by experts. However, it is fairly hard to acquire a large-scale dataset for COVID-19 with accurate labels in regard to the shortage of radiologists in clinics, the emergency of the disease, and etc. With a limited amount of samples, the performance of such methods is in question. Prior knowledge on the disease could aid in extracting useful features for the diagnosis, and thus alleviating the problem of lack of the labeled data. The level of the clinical and scientific understanding of COVID-19 is still immature, i.e., difficult to incorporate prior knowledge in developing a CT diagnostic system for COVID-19.

Several research efforts have been made to develop deep learning methods that can identify patients with COVID-19 in CT images [19]. Previous works can be roughly grouped into two types: 1) slice-level classification and 2) patient-level classification. Slice-level classification approaches conduct the classification either per CT image or per region in CT images. For example [20], proposed a deep learning framework for the classification of COVID-19, other pneumonia, and no pneumonia. [21] utilized an image processing technique to extract lung regions, a 3D CNN to select candidate regions, and a classification model to categorize the regions into COVID-19, Influenza-A-viral-penumonia, and irrelevant-to-infection. Many of patient-level classification approaches have adopted multiple neural networks for the diagnosis of COVID-19. For instance, two CNNs for lung/lobe segmentation and COVID-19 classification [22–24] and an attention-based 3D multiple instance network [25] and and a dual-sampling attention network [26] for COVID-19 diagnosis. Moreover [27], conducted the slice-level infection segmentation for COVID-19 and [28] utilized a deep learning method to quantify lung burden changes in patients with COVID-19 from serial CT scans.

Semi-supervised learning is an approach where both labeled and unlabeled data could be utilized in a cooperative manner [29]. The amount of unlabeled data keeps increasing in medical imaging. Semi-supervised learning provides a way to utilize the unlabeled data without the cost of data annotation, which is a bottleneck of the technical advances. Its effectiveness in medical imaging has recently been demonstrated in several applications, including cardiac segmentation [30], lung nodule classification [31], and Sclerosis lesion segmentation [32]. As for CT images of COVID-19, although the amount of the labeled data is insufficient, there exists a great deal of lung CT datasets available to the public. Such lung CT datasets may contain CT images obtained from healthy subjects and patients with various types of diseases, including lung cancer, pulmonary nodules, and etc. By utilizing both CT images from the COVID-19 dataset and lung CT dataset, the diagnostic system for COVID-19 could explore more diverse and complicated patterns of lesions in lung, leading to an improved ability of characterizing and analyzing CT images. Therefore, semi-supervised learning could be beneficial to the development of the diagnostic system for COVID-19.

Herein, we present a deep learning-based system for COVID-19 diagnosis in CT images. The system is built upon advanced deep convolutional neural networks (CNNs) to extract and characterize latent features of infected lesions in CT images and to detect COVID-19 infection. During training, both COVID-19 and public lung CT datasets are used to train the deep learning system in a semi-supervised fashion. To assess the importance of semi-supervised learning, we conduct a number of comparative experiments with supervised and semi-supervised learning. The experimental results demonstrate the effectiveness and efficiency of the proposed method in identifying COVID-19 in CT images.

The main contributions of our work are summarized as follows:

- We propose a semi-supervised learning-based deep neural network for COVID-19 diagnosis in CT images that can extensively explore a wide range of CT images with and without the ground truth label.
- We employ two COVID-19 CT datasets and three public lung CT datasets without COVID-19 to train, validate, and test the proposed diagnostic system in a rigorous manner.
- Utilizing the advanced deep learning techniques and semi-supervised learning, we achieve an accuracy of 99.83%, sensitivity of 0.9286, specificity of 0.9832, and positive predictive value (PPV) of 0.9192 in distinguishing COVID-19 from non-COVID-19 CT images. In the classification between COVID-19 and common pneumonia CT images, 96.98% accuracy, 0.9968 sensitivity, 0.9548 specificity, and 0.9248 PPV are obtained.

## Materials and methods

**Fig 1:**
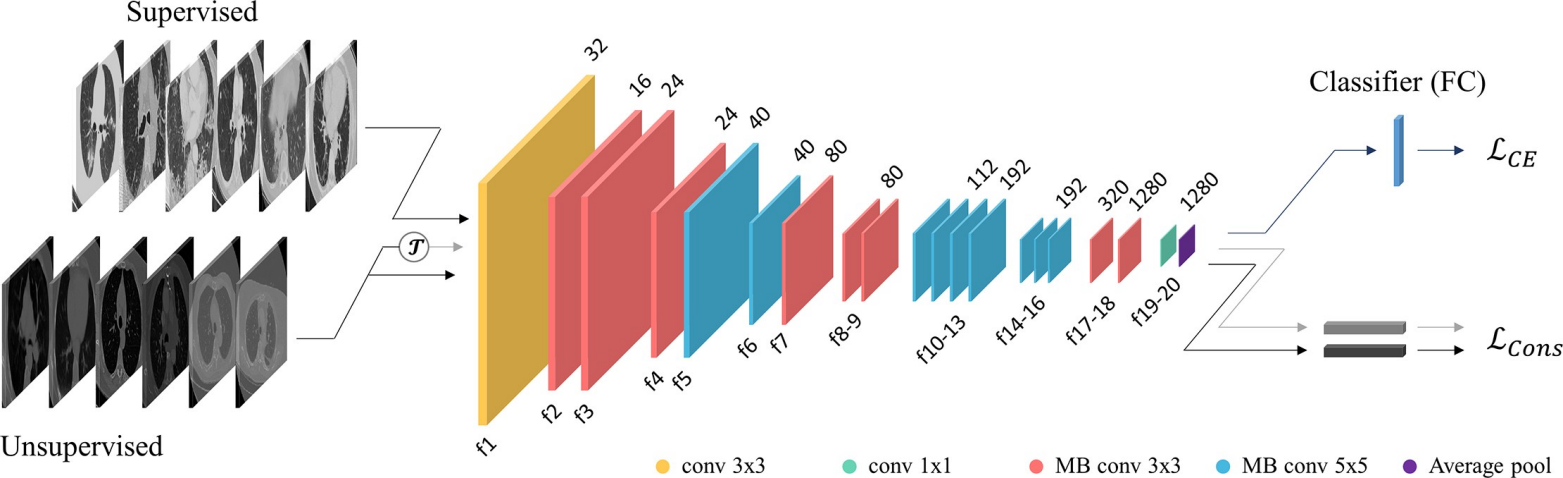
Overview of the proposed diagnostic system for COVID-19.

Fig 1 illustrates the proposed diagnostic system for COVID-19. The proposed method utilizes a CNN to identify lung CT images with COVID-19. The network is trained and tested using the labeled COVID-19 dataset in a supervised fashion. To improve the stability and precision of the network, additional public CT datasets as well as an unsupervised learning approach have been adopted. Suppose that $D={\left\{\left(x^{i},y^{i}\right)\right\}}_{i=1}^{N}$ is a set of *N* labeled CT images and $U={\left\{{\hat{x}}^{i}\right\}}_{i=1}^{M}$ is a set of *M* unlabeled CT images where *x*^*i*^ is the *i*th labeled CT image, *y*^*i*^∈{0,1} is the ground truth label, and ${\hat{x}}^{i}$ is the *i*th unlabeled CT image. A CNN *ψ* represents a mapping function *ψ*: *x*→*y*. *ψ* consists of *L* functions or stages $\psi ={\left\{f_{l};\theta_{l}\right\}}_{l=1}^{L}$ where *f*_*l*_ and *θ*_*l*_ indicate the *l*th operation and the corresponding parameters, respectively. We also note that *ψ*_*m*_ represents the set of the first *m* stages, i.e., $\psi_{m}={\left\{f_{l};\theta_{l}\right\}}_{l=1}^{m}$.

### Network architecture

**Fig 2:**
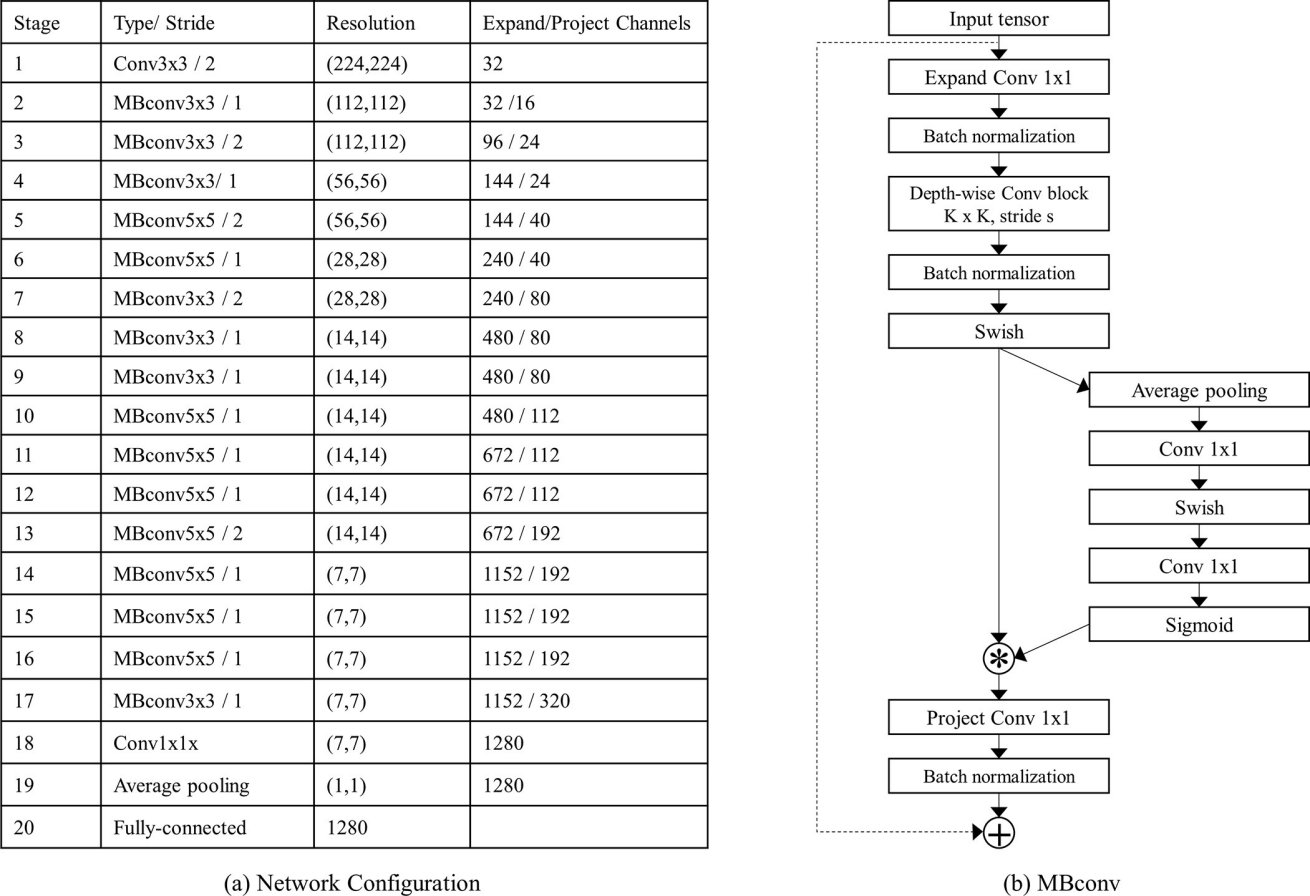
Network architecture. (a) The details of the network architecture and (b) MBconv block are shown.

The proposed network adopts the architecture of efficientNet-b0 [33]. The network is composed of 20 stages (Fig 2). The first stage (*f*_1_) simply conducts a convolution operation with a kernel size of 3x3. From *f*_3_ to *f*_17_, each stage utilizes a mobile inverted bottleneck convolution (MBConv) block, which consists of a series of an expand point-wise convolution (1x1 convolution that expands the number of channels), a depth-wise separable convolution (a single convolution per channel), and a project point-wise convolution (1x1 convolution to project features to a low-dimensional space). *f*_2_ omits the expand point-wise convolution. Each *f*_*l*_ BN block has a shortcut connection between the input and the output of the block except *f*_3_, *f*_5_, *f*_7_, and *f*_13_ blocks. A squeeze-and-excitation scheme is also utilized to incorporate the channel-wise interdependencies. The squeeze-and-excitation scheme aims to aggregate the global information of feature maps via a global average pooling (squeeze) and to recalibrate the feature maps via a combination of a sigmoid function, RELU function, and convolutions (excitation). The last stage, including a 1x1 convolution layer, an average pooling layer, and a fully-connected layer, conducts the classification. Swish function, instead of ReLU, is adopted as the activation function of the network. We can divide the whole network into two parts–a feature extractor (up to 19th stage) and a classifier (20th stage).

### Loss functions

The set of the parameters of the proposed network ${\left\{\theta_{l}\right\}}_{l=1}^{L}$ is optimized by utilizing the loss function L defined as:

$$L=L_{CE}+\lambda L_{Cons}$$ (1)

where $L_{CE}$ is a standard cross entropy loss function for supervised learning, $L_{Cons}$ is a consistency loss function for unsupervised learning, and *λ* is a weighting factor for $L_{Cons}$. For $L_{CE}$, only the labeled data is utilized. As for $L_{Cons}$, both labeled and unlabeled data can be used, ignoring the ground truth label for the labeled data.

$L_{CE}$ is adopted to calculate the total entropy between *p* and *y* as follows:

$$L_{CE}(p,y)=-\frac{1}{N}\sum_{i=1}^{N}\left(y^{i}\log p^{i}+(1-y^{i})\log\left(1-p^{i}\right)\right)$$ (2)

where *p*^*i*^ is the output of the CNN *ψ* as given the input image *x*^*i*^, i.e., *p*^*i*^ = *ψ*(*x*^*i*^; *θ*_1_,…,*θ*_*L*_). During training, $L_{CE}$ is minimized using the labeled CT images in a supervised manner.

To improve the consistency and robustness of the CNN *ψ*, we force the CNN *ψ* to be invariant to a perturbation in an input image. Given an unlabeled input image ${\hat{x}}^{i}$, *ψ*_*L*−1_, i.e., a feature extract, produces a high-dimensional feature vector $o^{i}=\psi_{L-1}({\hat{x}}^{i};\theta_{1},\ldots ,\theta_{L-1})\in R^{m}$. Suppose that there exists a set of augmentation functions $T=\{t_{i}:i=1,\ldots ,k\}$ that can deform the image ${\hat{x}}^{i}$ while maintaining its intrinsic structural and functional characteristics where *k* is the number augmentation functions. Then, ${\hat{x}}^{i}$ and $T({\hat{x}}^{i})$ carry the same or similar latent information. There should be no or minimal difference between the two high-dimensional feature vectors $\psi_{L-1}({\hat{x}}^{i};\theta_{1},\ldots ,\theta_{L-1})$ and $\psi_{L-1}\left(T({\hat{x}}^{i});\theta_{1},\ldots ,\theta_{L-1}\right)$. We define a consistency loss as:

$$L_{Cons}(x)=-\frac{1}{M}\sum_{i=1}^{M}\left|\psi_{L-1}({\hat{x}}^{i};\theta_{1},\ldots ,\theta_{L-1})-\psi_{L-1}(T({\hat{x}}^{i});\theta_{1},\ldots ,\theta_{L-1})\right|.$$ (3)

### Implementation details

Given an input image, we resize its spatial size to 256 x 256 pixels. Then, a number of data augmentation techniques are applied to transform the shape of the image as follows: 1) a random scaling in a range of [0.8, 12]; 2) a random translation within 1% of the width and height of the image; 3) a random shearing in a range of [-5°, 5°]; 4) a random horizontal flip with a probability of 0.5. Following the center-crop of size 224 x 224 pixels, one of three operations–a Gaussian blur, a median blur, and an additive Gaussian noise–are randomly selected and applied to make slight changes in the intensities of the image: 1) a Gaussian blur uses a Gaussian kernel with *σ* whose value is randomly chosen in a range of [0.0, 3.0]; 2) a median blur has a random kernel size with one of 3, 4, and 5; 3) an additive Gaussian noise is added to each channel that is randomly sampled from a Gaussian probability density function with *μ* = 0 and *σ* is a random value in a range of [0.0, 12.75]. Finally, the lightness of the image is adjusted by adding a random value in a range of [–20, 20] per pixel.

We use Adam optimizer with a mini-batch size of 12 and a weight decay of 0. The number of training epochs is set to 50 and the learning rate is set to 1.0e-4. Dropout layers are implemented with the probability of 0.2. The weighting factor *λ* is set to 0.3. The networks are implemented in PyTorch.

### Dataset

This study employs two CT datasets of COVID-19. The first dataset (*Data*^*COV*^), acquired from CT COVID-19 challenge (https://covid-ct.grand-challenge.org), contains 349 CT images from 216 COVID-19 patients and 463 CT images from 55 non-COVID-19 patients. The second dataset (*Data*^*COV-CV*^), obtained from the COVID-19 and common pneumonia chest CT dataset, consists of 416 CT scans from 206 COVID-19 patients and 412 CT scans from 412 patients with common pneumonia (CP), including viral pneumonia, bacterial pneumonia, and fungal pneumonia [34].

*Data*^*COV*^ is split into three disjoint sets by the challenge organizer, including a training set (*Train*^*COV*^), validation set (*Validation*^*COV*^), and test set (*Test*^*COV*^). The training set, validation set, and test set consists of 425 images (191 COVID-19, 234 non-COVID-19), 118 images (60 COVID-19, 58 non-COVID-19), and 203 images (98 COVID-19, 105 non-COVID-19), respectively. The dataset is collected from a number of online sources, including medRxiv, bioRxiv, NEJM, JAMA, Lancet, and etc. Similarly, *Data*^*COV-CP*^ is divided into a training set (*Train*^*COV-CP*^; 328 COVID-19 and 320 CP CT scans), validation set (*Validation*^*COV-CP*^; 46 COVID-19 and 42 CP CT scans), and test set (*Test*^*COV-CP*^; 42 COVID-19 and 40 CP CT scans).

We extend the two COVID-19 CT datasets by introducing three publicly available lung CT datasets:

1. Lung CT Segmentation Challenge 2017 (LCTSC): This dataset is composed of CT scans from 60 patients that are collected from three institutions. CT scans may include inflated and collapsed, fibrotic and emphysematic lungs, but lung tumor is most likely excluded. We split this dataset into the training set and the test set, of which each contains 30 CT scans.
2. Reference Image Database to Evaluate Therapy Response (RIDER): This dataset consists of CT scans from 32 patients with non-small cell lung cancer. Two CT scans are collected from each patient with 15-minutes time gap. Both CT scans are utilized in this study and divided into the training set with 16 patients and the test set with 16 patients. The training set has 32 CT scans and the test set has 31 scans since one CT scan is missed for one patient.
3. LUNGx SPIE-AAPM-NCI Lung Nodule Classification Challenge (LUNGx): LUNGx contains 70 CT scans from patients with malignant and benign nodules. 70 CT scans are split into 34 CT scans (17 benign and 17 malignant nodules) for training and 36 CT scans (18 benign and 18 malignant nodules) for test.

**Fig 3:**
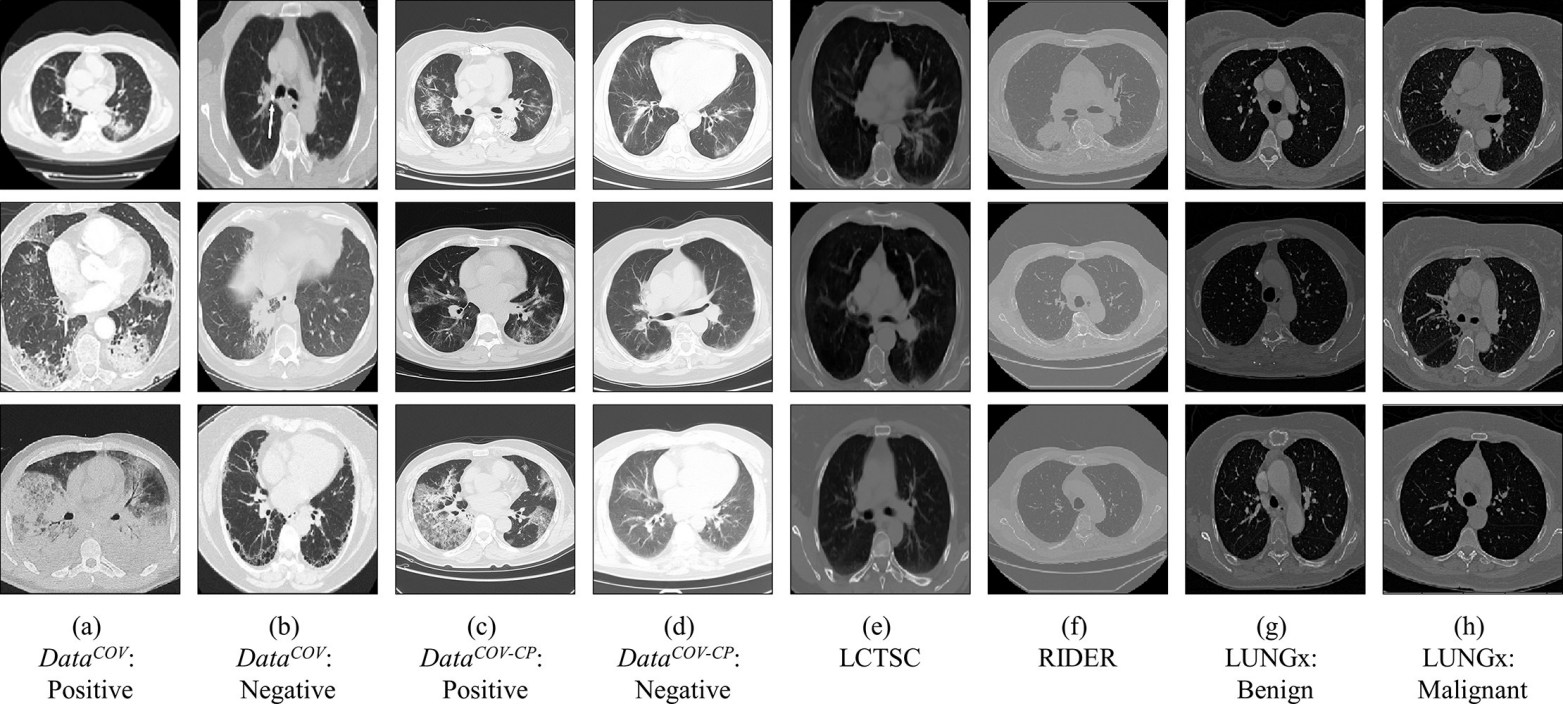
Exemplary CT images of the COVID-19 and public datasets.

For these three public, called as *Data*^*PUB*^, we manually select a number of slices that can clearly visualize a lung. In the training set (*Train*^*PUB*^), we randomly select 5 slices per scan for efficient training and balance with *Data*^*COV*^. In the test set (*Test*^*PUB*^), all the slices are utilized. The details of the training set, validation set, and test set are available in Table 1. Some exemplary CT images are shown in Fig 3.

**Table 1:** Details of the datasets.

Dataset	Type	Training set	Validation set	Test set
*Data*^*COV*^	COVID-19	191	60	98
	non-COVID-19	115	28	52
*Data*^*COV-CP*^	COVID-19	35299	3719	2789
	Common Pneumonia	45558	4754	5004
*Data*^*PUB*^	LCTSC	160	-	1577
	RIDER	170	-	3216
	LUNGx	786	-	4099
Total		82279	8561	16835

Each number represents the number of slices

### Experimental setup

To evaluate the proposed diagnostic system for COVID-19, we conduct two types of experiments. The first type of the experiments, employing *Data*^*COV*^ and *Data*^*PUB*^, is to test if the proposed system could distinguish COVID-19 CT slices from non-COVID-19 CT slices. The second type of the experiments, including *Data*^*COV-CP*^ and *Data*^*PUB*^, attempts to assess whether the proposed system could discriminate between COVID-19 CT slices and CP CT slices.

For the first type of the experiments, we train the proposed diagnostic system using both *Train*^*COV*^ and *Train*^*PUB*^, select the best model based upon the performance on *Validation*^*COV*^, and test the best model on *Test*^*COV*^ and *Test*^*PUB*^. During the training phase, *Train*^*COV*^ is used to compute both $L_{CE}$ (as labeled data) and $L_{Cons}$ (as unlabeled data) and *Train*^*PUB*^ is used to calculate $L_{Cons}$ only. In the testing phase, all the slices from *Test*^*PUB*^ are treated as negative. To further assess the effectiveness of semi-supervised learning, we conduct a series of comparative experiments. First, the diagnostic system is trained in a supervised manner using *Train*^*COV*^ only. Second, both *Train*^*COV*^ and *Train*^*PUB*^ are used for supervised learning only. Third, we train the diagnostic system using *Train*^*COV*^ in a supervised and unsupervised fashion. Last, both *Train*^*COV*^ and *Train*^*PUB*^ are used for supervised and unsupervised training. In summary, the first two experiments are to compare semi-supervised learning with supervised-learning. The latter two experiments are to investigate the relationship between semi-supervised learning and the datasets. For all the experiments, the best model is chosen using *Validation*^*COV*^ and is tested on *Test*^*COV*^ and *Test*^*PUB*^.

Similarly, *Data*^*COV-CP*^ and *Data*^*PUB*^ are used to train the diagnostic system, select the best model, and test on the test set in the second type of the experiments. For the training purpose, $L_{CE}$ is computed using *Train*^*COV-CP*^ and $L_{Cons}$ is calculated using both *Train*^*COV-CP*^ and *Train*^*PUB*^. In the testing phase, *Test*^*PUB*^ is omitted since it includes CT scans from healthy subjects and patients with other diseases such as cancer. As for the comparative experiments, we conduct the first and third comparative experiments since *Test*^*PUB*^ cannot be adopted as negative sample in this type of experiments.

## Results

### Detection of COVID-19 in CT images

In distinguishing COVID-19 CT slices from non-COVID-19 slices, the proposed semi-supervised learning method obtained an overall accuracy of 99.83%, sensitivity of 0.9286, specificity of 0.9991, and positive predictive value (PPV) of 0.9192 on *Test*^*COV*^ and *Test*^*PUB*^ as shown in Table 2. In regard to the COVID-19 challenge dataset only (*Test*^*COV*^), 94.67% accuracy, 0.9286 sensitivity, 0.9808 specificity, and 0.9891 PPV were achieved. On the public dataset (*Test*^*PUB*^) where only negative (non-COVID-19) slices are included, the proposed method acquired 0.9835 specificity. Moreover, the proposed method was able to discriminate between COVID-19 CT slices and CP CT slices. The method achieved an accuracy of 97.32%, sensitivity of 0.9971, specificity of 0.9598, and PPV of 0.9326 on *Test*^*COV-CP*^ (Table 3).

**Table 2:** Classification results: COVID-19 vs. non-COVID-19 CT slices.

*Test*	$L_{CE}$	*Train*^*COV*^	*Train*^*COV*^	*Train*^*COV*^	*Train*^*COV*^ *& Train*^*PUB*^	*Train*^*COV*^ *& Train*^*PUB*^
	$L_{Cons}$	-	*Train*^*COV*^	*Train*^*COV*^ *& Train*^*PUB*^	-	*Train*^*COV*^ *& Train*^*PUB*^
*Test*^*COV*^ *& Test*^*PUB*^	ACC	99.18%	99.52%	99.83%	99.88%	99.89%
	Sens	0.8980	0.8980	0.9286	0.9490	0.9388
	Spec	0.9928	0.9963	0.9991	0.9993	0.9996
	PPV	0.5789	0.7273	0.9192	0.9394	0.9583
*Test*^*COV*^	ACC	89.33%	91.33%	94.67%	92.67%	93.33%
	Sens	0.8980	0.8980	0.9286	0.9490	0.9388
	Spec	0.8846	0.9423	0.9808	0.8846	0.9231
	PPV	0.9362	0.9670	0.9891	0.9394	0.9583
*Test*^*PUB*^	Spec	0.9935	0.9966	0.9992	1.0000	1.0000

ACC: accuracy, Sens: sensitivity, Spec: specificity, PPV: positive predictive value

**Table 3:** Classification results: COVID-19 vs. common pneumonia CT slices.

*Test*	$L_{CE}$	*Train*^*COV-CP*^	*Train*^*COV-CP*^	*Train*^*COV-CP*^
	$L_{Cons}$	-	*Train*^*COV-CP*^	*Train*^*COV-CP*^ *& Train*^*PUB*^
*Test*^*COV-CP*^	ACC	96.65%	96.98%	97.32%
	Sens	0.9975	0.9968	0.9971
	Spec	0.9492	0.9548	0.9598
	PPV	0.9163	0.9248	0.9326

ACC: accuracy, Sens: sensitivity, Spec: specificity, PPV: positive predictive value

### Comparative experiments: Supervised learning

In comparison to the COVID-19 detection model (first type of experiments), two models were built via supervised learning–one uses *Train*^*COV*^ only and the other uses both *Train*^*COV*^ and *Train*^*PUB*^. Using *Train*^*COV*^ only, the performance of COVID-19 detection decreased by >0.6% in accuracy, >0.03 in sensitivity, >0.07 in specificity, and >0.44 in PPV on *Test*^*COV*^ and *Test*^*PUB*^. When it was tested on *Test*^*COV*^ only, the performance degradation was even severer; >5% in accuracy. >0.03 in sensitivity, and >0.09 in specificity. Utilizing unsupervised learning, the ability of the classification model was substantially improved, in particular for the detection of COVID-19 samples.

Using both *Train*^*COV*^ and *Train*^*PUB*^ in a supervised fashion, there was a marginal improvement in the overall classification performance; for instance, ~0.05% in accuracy, ~0.02 in sensitivity, and ~0.02 in PPV. However, the performance on *Test*^*COV*^ was inferior to the proposed semi-supervised method; 2%, ~0.1, and ~0.05 decrease in accuracy, specificity, and PPV, respectively, leading to a substantial increase in false positive cases.

As for the classification of COVID-19 vs. CP CT slices (second type of experiments), the similar observations were made with the first type of experiments. one supervised learning model was built on *Train*^*COV-CP*^ and compared to the semi-supervised learning model. Similar to the result of the COVID-19 detection, there was a performance drop by the supervised model such as >0.6% in accuracy, >0.01 in specificity, and >0.01 PPV.

### Comparative experiments: Semi-supervised vs. supervised learning

In distinguishing COVID-19 from non-COVID-19 CT slices, each of the supervised methods was compared to its semi-supervised counterpart. Using *Train*^*COV*^ in a semi-supervised manner, the accuracy, specificity, and PPV were improved by >0.3%, ~0.004, and ~0.15, respectively, on *Test*^*COV*^ and *Test*^*PUB*^, in comparison to the supervised method. On *Test*^*COV*^ only, 2%, >0.05, and >0.03 improvement in accuracy, specificity, and PPV, respectively, were observed. On *Test*^*PUB*^ only, the semi-supervised method showed better performance in the specificity by >0.03. Similarly, using both *Train*^*COV*^ and *Train*^*PUB*^, the semi-supervised method, by and large, outperformed the supervised counterpart. Moreover, we attained a performance gain by >0.03% in accuracy, >0.005 in specificity, and >0.008 in PPV as utilizing *Train*^*COV-CP*^ in a semi-supervised manner on *Test*^*COV-CP*^. These results demonstrate the effectiveness of semi-supervised learning in comparison to supervised learning for both classification tasks.

For both classification tasks, the semi-supervised method that uses either *Train*^*COV*^ or *Train*^*COV-CP*^ for both supervised and unsupervised learning was compared to the proposed semi-supervised method that additionally utilizes *Train*^*PUB*^ in an unsupervised manner, i.e., computing $L_{Cons}$. Using the extra *Train*^*PUB*^, the accuracy, sensitivity, specificity, and PPV were improved in all the experiments. Furthermore, adopting *Train*^*PUB*^ in a supervised learning as negative samples (first type of experiments), the classification performance was slightly improved in all the experiments except the accuracy, specificity, and PPV on *Test*^*COV*^.

## Discussions

A rapid, reliable, and accurate detection of COVID-19 has a direct bearing on the global healthcare due to the prevalence of the disease and its impact on the society. Although CT images have shown to be useful in detecting COVID-19, the limited understanding and lack of relevant COVID-19 data pose a difficulty in developing diagnostic tools. The proposed semi-supervised learning approach demonstrates an alternative manner to develop an accurate and robust diagnostic tool for COVID-19 that can utilize both COVID-19 CT images and non-COVID-19 CT images.

The proposed semi-supervised method was able to detect COVID-19 CT images with high accuracy. On the public CT images, including both healthy lungs and lungs with other types of diseases, the method was successfully applied. Even though the pubic CT images miss COVID-19, the good classification performance guarantees that there would be no or minimal misdiagnosis on healthy subjects with respect to COVID-19, lowering the burden and cost of healthcare. The additive value of the proposed method will be apparent as it is implemented as a pre-screening mechanism. For instance, the proposed method will mis-classify <1% of non-COVID-19 CT images as positive (at specificity of 0.9991), significantly lowering the workload, whereas 7.14% of COVID-19 CT images (at sensitivity of 0.9286) will be missed and ~8% of the positive CT images will include non-COVID-19 CT images (at PPV of 0.9192). In regard to CP, <1% of COVID-19 CT images will be missed, while <5% of the CP CT images will make it through the screening and these CP CT images will occupy <8% of the positive CT images. We note that a direction comparison with the RT-PCT test may be misleading since the proposed method is applied to a per CT slice not per patient. Each CT scan per patient includes a number of CT images and a lesion may present in multiple CT images. The chance of mis-classifying multiple CT images, i.e., missing COVID-19 patients, will be likely lower than what reported here.

In comparison to the supervised methods, the effectiveness and efficiency of the proposed semi-supervised method have been demonstrated. The supervised method, trained on either *Train*^*COV*^ or *Train*^*COV-CP*^, was inferior to the proposed method. The proposed semi-supervised method is capable of not only improving the diagnostic accuracy on COVID-19 CT images but also lowering the rate of misdiagnosis rate for non-COVID-19 CT images. However, the supervised method, utilizing both the COVID-19 dataset (*Train*^*COV*^) and public dataset (*Train*^*PUB*^), was generally superior to the proposed semi-supervised method by a small margin. Since supervised learning requires labeled data and labeling is, in general, costly and time-consuming, it is hard to further extend the approach. There is a trade-off between the performance and the labeling cost. The proposed semi-supervised method allows us to improve the classification performance as close as the supervised method without the high cost of data labeling.

The effect of semi-supervised learning was not dependent on the training dataset. For both supervised methods–one uses *Train*^*COV*^ only and the other uses both *Train*^*COV*^ and *Train*^*PUB*^, the adoption of unsupervised learning approach, i.e., calculating $L_{Cons}$, gives rise to the improvement in the classification performance in general. This indicates that the proposed method enhances the utility of the available dataset, leading to an improved performance in disease diagnosis.

In semi-supervised learning, the classification performance varies depending on the training strategy. Although the same dataset (*Train*^*COV*^ or *Train*^*COV-CP*^) is used for supervised training, the addition of the extra dataset (*Train*^*PUB*^) for unsupervised training results in an improved ability of COVID-19 detection, emphasizing the importance of the amount and diversity of the dataset in unsupervised training. Furthermore, we gained a slight performance gain by adding the labeled dataset (*Train*^*PUB*^) in supervised training while the training dataset (*Train*^*COV*^ and *Train*^*PUB*^) remains the same for unsupervised training. As we discussed above, this requires an extensive labeling for all available datasets, which is impractical and inefficient in many of medical imaging domains.

This study has several limitations. A limited amount of CT images with COVID-19 were utilized in this study. The reported performance should be understood with respect to the characteristics of the dataset used here. A follow-up study should be conducted to further validate the accuracy and utility of the proposed method. The proposed method detects COVID-19 per CT slice. Localization of COVID-19 in a CT slice could aid in analyzing the disease and the classification results. The improved learning ability via the proposed semi-supervised approach should be applicable to the localization of COVID-19 on CT images.

## Conclusions

Herein, we propose an advanced, diagnostic tool for COVID-19 in CT images. The approach adopts a semi-supervised learning scheme that exploits both supervised and unsupervised learning. The experimental results show that the proposed semi-supervised method is able to detect CT images with COVID-19 in an accuracy and robust manner. Adopting the public CT images in an unsupervised fashion, in particular, the proposed method achieves a substantial performance gain, indicating that the proposed method is able to utilize the non-COVID-19 CT images for an improved COVID-19 diagnosis. The approach is generic and can be applicable to other types of diseases and datasets in other domains to enhance the learning capability of the classification model and to extend the utility of the dataset.

## References

[1] Zhu N, Zhang D, Wang W, Li X, Yang B, Song J, et al. A Novel Coronavirus from Patients with Pneumonia in China, 2019. New England Journal of Medicine. 2020;382(8):727–33. 10.1056/NEJMoa2001017 . 31978945 PMC7092803

[2] Andersen KG, Rambaut A, Lipkin WI, Holmes EC, Garry RF. The proximal origin of SARS-CoV-2. Nature Medicine. 2020;26(4):450–2. 10.1038/s41591-020-0820-9 32284615 PMC7095063

[3] Wang W, Tang J, Wei F. Updated understanding of the outbreak of 2019 novel coronavirus (2019-nCoV) in Wuhan, China. J Med Virol. 2020;92(4):441–7. Epub 2020/02/12. 10.1002/jmv.25689 . 31994742 PMC7167192

[4] Wang C, Horby PW, Hayden FG, Gao GF. A novel coronavirus outbreak of global health concern. The Lancet. 2020;395(10223):470–3. 10.1016/S0140-6736(20)30185-9 31986257 PMC7135038

[5] Lee JY, Kim HA, Huh K, Hyun M, Rhee JY, Jang S, et al. Risk Factors for Mortality and Respiratory Support in Elderly Patients Hospitalized with COVID-19 in Korea. J Korean Med Sci. 2020;35(23):e223–e. 10.3346/jkms.2020.35.e223 . 32537957 PMC7295602

[6] Ai T, Yang Z, Hou H, Zhan C, Chen C, Lv W, et al. Correlation of Chest CT and RT-PCR Testing for Coronavirus Disease 2019 (COVID-19) in China: A Report of 1014 Cases. Radiology. 2020;296(2):E32–E40. 10.1148/radiol.2020200642 . 32101510 PMC7233399

[7] Song F, Shi N, Shan F, Zhang Z, Shen J, Lu H, et al. Emerging 2019 Novel Coronavirus (2019-nCoV) Pneumonia. Radiology. 2020;295(1):210–7. 10.1148/radiol.2020200274 32027573 PMC7233366

[8] Li Y, Xia L. Coronavirus disease 2019 (COVID-19): role of chest CT in diagnosis and management. American Journal of Roentgenology. 2020;214(6):1280–6. 10.2214/AJR.20.22954 32130038

[9] Bernheim A, Mei X, Huang M, Yang Y, Fayad ZA, Zhang N, et al. Chest CT Findings in Coronavirus Disease-19 (COVID-19): Relationship to Duration of Infection. Radiology. 2020;295(3):200463. 10.1148/radiol.2020200463 32077789 PMC7233369

[10] Huang P, Liu T, Huang L, Liu H, Lei M, Xu W, et al. Use of Chest CT in Combination with Negative RT-PCR Assay for the 2019 Novel Coronavirus but High Clinical Suspicion. Radiology. 2020;295(1):22–3. 10.1148/radiol.2020200330 . 32049600 PMC7233360

[11] Xie X, Zhong Z, Zhao W, Zheng C, Wang F, Liu J. Chest CT for Typical Coronavirus Disease 2019 (COVID-19) Pneumonia: Relationship to Negative RT-PCR Testing. Radiology. 2020;296(2):E41–E5. 10.1148/radiol.2020200343 32049601 PMC7233363

[12] Shen D, Wu G, Suk H-I. Deep Learning in Medical Image Analysis. Annual Review of Biomedical Engineering. 2017;19(1):221–48. 10.1146/annurev-bioeng-071516-044442 . 28301734 PMC5479722

[13] Shin H, Roth HR, Gao M, Lu L, Xu Z, Nogues I, et al. Deep Convolutional Neural Networks for Computer-Aided Detection: CNN Architectures, Dataset Characteristics and Transfer Learning. IEEE Transactions on Medical Imaging. 2016;35(5):1285–98. 10.1109/TMI.2016.2528162 26886976 PMC4890616

[14] Chilamkurthy S, Ghosh R, Tanamala S, Biviji M, Campeau NG, Venugopal VK, et al. Deep learning algorithms for detection of critical findings in head CT scans: a retrospective study. The Lancet. 2018;392(10162):2388–96. 10.1016/S0140-6736(18)31645-3 30318264

[15] Ting DSW, Cheung CY, Lim G, Tan GSW, Quang ND, Gan A, et al. Development and Validation of a Deep Learning System for Diabetic Retinopathy and Related Eye Diseases Using Retinal Images From Multiethnic Populations With Diabetes. Jama. 2017;318(22):2211–23. Epub 2017/12/14. 10.1001/jama.2017.18152 29234807 PMC5820739

[16] Ehteshami Bejnordi B, Veta M, Johannes van Diest P, van Ginneken B, Karssemeijer N, Litjens G, et al. Diagnostic Assessment of Deep Learning Algorithms for Detection of Lymph Node Metastases in Women With Breast Cancer. Jama. 2017;318(22):2199–210. Epub 2017/12/14. 10.1001/jama.2017.14585 29234806 PMC5820737

[17] Graham S, Vu QD, Raza SEA, Azam A, Tsang YW, Kwak JT, et al. Hover-Net: Simultaneous segmentation and classification of nuclei in multi-tissue histology images. Medical Image Analysis. 2019;58:101563. 10.1016/j.media.2019.101563 31561183

[18] LeCun Y, Bengio Y, Hinton G. Deep learning. Nature. 2015;521(7553):436–44. Epub 2015/05/29. 10.1038/nature14539 . 26017442

[19] Shi F, Wang J, Shi J, Wu Z, Wang Q, Tang Z, et al. Review of Artificial Intelligence Techniques in Imaging Data Acquisition, Segmentation and Diagnosis for COVID-19. IEEE Reviews in Biomedical Engineering. 2020:1-. 10.1109/RBME.2020.2987975 32305937

[20] Ko H, Chung H, Kang WS, Kim KW, Shin Y, Kang SJ, et al. COVID-19 Pneumonia Diagnosis Using a Simple 2D Deep Learning Framework With a Single Chest CT Image: Model Development and Validation. J Med Internet Res. 2020;22(6):e19569. 10.2196/19569 32568730 PMC7332254

[21] Xu X, Jiang X, Ma C, Du P, Li X, Lv S, et al. A Deep Learning System to Screen Novel Coronavirus Disease 2019 Pneumonia. Engineering. 2020. 10.1016/j.eng.2020.04.010 32837749 PMC7320702

[22] Wang S, Zha Y, Li W, Wu Q, Li X, Niu M, et al. A Fully Automatic Deep Learning System for COVID-19 Diagnostic and Prognostic Analysis. European Respiratory Journal. 2020:2000775. 10.1183/13993003.00775-2020 32444412 PMC7243395

[23] Wang J, Bao Y, Wen Y, Lu H, Luo H, Xiang Y, et al. Prior-Attention Residual Learning for More Discriminative COVID-19 Screening in CT Images. IEEE Transactions on Medical Imaging. 2020;39(8):2572–83. 10.1109/TMI.2020.2994908 32730210

[24] Wang X, Deng X, Fu Q, Zhou Q, Feng J, Ma H, et al. A Weakly-Supervised Framework for COVID-19 Classification and Lesion Localization From Chest CT. IEEE Transactions on Medical Imaging. 2020;39(8):2615–25. 10.1109/TMI.2020.2995965 33156775

[25] Han Z, Wei B, Hong Y, Li T, Cong J, Zhu X, et al. Accurate Screening of COVID-19 Using Attention-Based Deep 3D Multiple Instance Learning. IEEE Transactions on Medical Imaging. 2020;39(8):2584–94. 10.1109/TMI.2020.2996256 32730211

[26] Ouyang X, Huo J, Xia L, Shan F, Liu J, Mo Z, et al. Dual-Sampling Attention Network for Diagnosis of COVID-19 from Community Acquired Pneumonia. IEEE Transactions on Medical Imaging. 2020. 10.1109/TMI.2020.2995508 32730212

[27] Fan D-P, Zhou T, Ji G-P, Zhou Y, Chen G, Fu H, et al. Inf-Net: Automatic COVID-19 Lung Infection Segmentation from CT Images. IEEE Transactions on Medical Imaging. 2020. 10.1109/TMI.2020.2996645 32730213

[28] Huang L, Han R, Ai T, Yu P, Kang H, Tao Q, et al. Serial Quantitative Chest CT Assessment of COVID-19: Deep-Learning Approach. Radiology: Cardiothoracic Imaging. 2020;2(2):e200075. 10.1148/ryct.2020200075 33778562 PMC7233442

[29] van Engelen JE, Hoos HH. A survey on semi-supervised learning. Machine Learning. 2020;109(2):373–440. 10.1007/s10994-019-05855-6

[30] Bai W, Oktay O, Sinclair M, Suzuki H, Rajchl M, Tarroni G, et al., editors. Semi-supervised Learning for Network-Based Cardiac MR Image Segmentation2017; Cham: Springer International Publishing.

[31] Xie Y, Xia Y, Zhang J, Song Y, Feng D, Fulham M, et al. Knowledge-based Collaborative Deep Learning for Benign-Malignant Lung Nodule Classification on Chest CT. IEEE Trans Med Imaging. 2019;38(4):991–1004. Epub 2018/10/20. 10.1109/TMI.2018.2876510 . 30334786

[32] Baur C, Albarqouni S, Navab N, editors. Semi-supervised Deep Learning for Fully Convolutional Networks2017; Cham: Springer International Publishing.

[33] Tan M, Le Q, editors. EfficientNet: Rethinking Model Scaling for Convolutional Neural Networks. International Conference on Machine Learning; 2019.

[34] Yan T, Wong PK, Ren H, Wang H, Wang J, Li Y. Automatic distinction between covid-19 and common pneumonia using multi-scale convolutional neural network on chest ct scans. Chaos, Solitons & Fractals. 2020;140:110153. 10.1016/j.chaos.2020.110153 32834641 PMC7381895

